# Development and implementation of nested single‐nucleotide polymorphism (SNP) assays for breeding and genetic research applications

**DOI:** 10.1002/tpg2.20491

**Published:** 2024-07-22

**Authors:** Qijian Song, Charles Quigley, Ruifeng He, Dechun Wang, Henry Nguyen, Carrie Miranda, Zenglu Li

**Affiliations:** ^1^ USDA‐ARS, Soybean Genomics & Improvement Laboratory Beltsville Maryland USA; ^2^ Department of Plant, Soil and Microbial Sciences Michigan State University East Lansing Michigan USA; ^3^ Molecular Genetics and Soybean Genomics Laboratory, Division of Plant Science and Technology University of Missouri Columbia Missouri USA; ^4^ Department of Plant Sciences North Dakota State University Fargo North Dakota USA; ^5^ Institute of Plant Breeding, Genetics and Genomics/Department of Crop and Soil Sciences University of Georgia Athens Georgia USA

## Abstract

SoySNP50K and SoySNP6K are commonly used for soybean (*Glycine max*) genotyping. The SoySNP50K assay has been used to genetically analyze the entire USDA Soybean Germplasm Collection, while the SoySNP6K assay, containing a subset of 6000 single‐nucleotide polymorphisms (SNPs) from SoySNP50K, has been used for quantitative trait loci mapping of different traits. To meet the needs for genomic selection, selection of parents for crosses, and characterization of breeding populations, especially early selection of ideal offspring from thousands of lines, we developed two assays, SoySNP3K and SoySNP1K, containing 3072 and 1252 SNPs, respectively, based on SoySNP50K and SoySNP6K mark sets. These two assays also contained the trait markers reported or contributed by soybean breeders. The SNPs in the SoySNP3K are a subset from SoySNP6K, while the SNPs in the SoySNP1K are a subset from SoySNP3K. These SNPs were chosen to reduce the SNP number in the large linkage blocks while capturing as much of the haplotype diversity as possible. They are highly polymorphic and of high quality. The mean minor allele frequencies of the SNPs in the southern and northern US elites were 0.25 and 0.27 for SoySNP3K, respectively, and 0.29 and 0.33 for SoySNP1K. The selected SNPs are a valuable source for developing targeted amplicon sequencing assay or beadchip assay in soybean. SoySNP3K and SoySNP1K assays are commercialized by Illumina Inc. and AgriPlex Genomics, respectively. Together with SoySNP50K and SoySNP6K, a series of nested assays with different marker densities will serve as additional low‐cost genomic tools for genetic, genomic, and breeding research.

## Core Ideas

- Two assays containing SoySNP3K and SoySNP1K single‐nucleotide polymorphisms (SNPs) and markers associated with soybean traits were developed.
- SNPs in the assays are highly polymorphic among US Elites and well cover genome haplotype blocks.
- The assays are valuable resource for soybean genetic research and breeding applications.
- These assays are commercialized and used by soybean researchers.

**Abbreviations**
1K: SoySNP1K
3K: SoySNP3K
50K: SoySNP50K
6K: SoySNP6K
MAF: minor allele frequency
QTL: quantitative trait loci
SNP: single‐nucleotide polymorphism
WGS: whole genome sequence

## 1 INTRODUCTION

High‐throughput genotyping is an essential process for quantitative trait loci (QTL) mapping, genomic prediction, breeding selection, and variety property protection. Song et al. (2013) developed the first high‐throughput single‐nucleotide polymorphism (SNP) assay, SoySNP50K (50K), containing 42,509 SNPs in soybean after analyzing the whole genome sequence (WGS) of a diverse set of cultivated and wild soybean accessions. About forth‐fifth of the SNPs targeted euchromatin regions and one‐fifth heterochromatin regions of 20 soybean chromosomes, consistent with the recombination rate of heterochromatin regions being only one‐fifth that of euchromatin regions (Song et al., 2013). The 50K assay was successfully used to genotype the entire USDA Soybean Germplasm Collection containing 18,489 *Glycine max* and 1160 *Glycine soja* accessions and many bi‐parental populations (Song et al., 2015; Song et al., 2016). The 50K dataset deposited at Soybase (https://soybase.org/snps/) has been used by many soybean laboratories in the United States to map genes controlling diseases (McDonald et al., 2023; Vuong et al., 2015; Wen et al., 2014; Zhang, Singh, et al., 2015), insects (Bhusal et al., 2017; Chang & Hartman, 2017), stresses (Dhanapal et al., 2015; Do et al., 2019; Ray et al., 2015), productivity (Ravelombola et al., 2021), seed quality(Bandillo et al., 2015; Hwang et al., 2014), seed composition (Sung et al., 2021; Vaughn et al., 2014; Zeng et al., 2014), and morphological traits (Zhang, Song, et al., 2015; Zhang et al., 2016) via QTL linkage mapping or genome‐wide association analysis approach, to characterize genetic heterogeneity within soybean accessions (Mihelich et al., 2020), to construct high‐resolution genetic linkage maps for improving the soybean genome sequence assembly (Song et al., 2016) and to study soybean domestication (Wen et al., 2015), etc. Based on the 50K dataset, Song et al. (2020) analyzed the extent of linkage disequilibrium in each chromosome of the cultivated soybean and delineated soybean genome‐wide haplotype blocks. This information along with other factors such as SNP polymorphism and genotyping quality were used to develop SoySNP6K (6K) assay, which was commercialized by Illumina Inc. (http://www.illumina.com/areas‐of‐interest/agrigenomics/consortia.html). The 6K SNP assay contained a subset of 6000 SNPs from the 50K SNP assay, which has been shown by soybean researchers to be useful and efficient in mapping QTL/genes controlling traits, especially in bi‐parental recombinant inbred line (RILs) populations, such as resistance to frogeye leaf spot (McDonald et al., 2023), stem canker (Menke et al., 2023), sudden death syndrome (Lightfoot et al., 2018; Wen et al., 2014), aphids (Bhusal et al., 2017; Zhang et al., 2017), *Phytophthora sojae*, *Pythium irregulare*, and *Fusarium graminearum* (Stasko et al., 2016), tolerance to salt (Do et al., 2018), waterlogging (Ye et al., 2018), iron deficiency chlorosis (Merry et al., 2019), nitrogen fixation (Huo et al., 2019), seed isoflavone content (Li et al., 2018), protein content (Souza et al., 2023), and yield (Ye et al., 2018). The assay has also been used for plant variety protection (Achard et al., 2020) and performing genomic selection (Ma et al., 2016).

Despite the success of the 50K and 6K assays, breeders also need assays with lower marker density and lower cost for selection of parents for crossing, analysis of pedigree among germplasm, and especially early‐generation breeding selection of desirable offspring, because thousands of breeding lines will be evaluated in the process. But 50K or 6K assays provided too much detail, resulting in increased expense. In addition, markers associated with traits or genes controlling seed quality, and biotic and abiotic stress tolerance have been reported. Incorporating these trait markers, especially those associated with biotic and abiotic stresses into the SNP assays, will greatly facilitate selection of these traits in the early generations to accelerate breeding cycles by reducing the time and cost of field and greenhouse testing.

The genomic positions of 50K SNPs in Williams 82 WGS assemblies Glyma1.01 (v1) (Schmutz et al., 2010) and Wm82a2v1 (v2) were reported previously (Song et al., 2015; Song et al., 2016). With the advent of the soybean WGS, the location of these SNPs in the latest assembly Wm82a4.1 (v4) needs to be updated. This information will help users locate the SNPs in the soybean genome and identify candidate genes or develop markers for fine mapping at specific regions.

Our objective was to develop efficient SNP assays, SoySNP3K (3K) with a core set of approximately 3000 SNPs from 6K and SoySNP1K (1K) with approximately 1000 SNPs from 3K that could be used for soybean genetic studies and breeding line selection in breeding programs. The 3K and 1K assays, together with 50K and 6K, would form a series of nested SNP assays that will meet soybean community's needs for assays of different marker density to achieve their research goals. In addition, since the USDA Soybean Germplasm Collection's 50K dataset is available, and many populations have been genotyped using 6K, datasets generated using 3K or 1K assays can be compared to or combined with the datasets that already exist to mine alleles across germplasm or breeding programs. Another goal was to update 50K in the latest WGS assembly Wm82a4.1 (Valliyodan et al., 2019), including 6K, 3K, and 1K SNP sites.

## 2 MATERIALS AND METHODS

### 2.1 SNP analysis of soybean germplasm

DNA extracted from the seeds of 18,489 cultivated and 1161 wild soybean accessions available from the USDA Soybean Germplasm Collection were genotyped with the high‐throughput 50K Beadchip assay (Song et al., 2013; Song et al., 2015). The collection included a total of 562 elites that were released in North America, labeled as “Developed/Modern” in the GRIN (https://npgsweb.ars‐grin.gov/gringlobal/search). The 50K dataset, including North American elites genotyped using 42,509 SNPs was described by Song et al. (2015) and deposited at SoyBase (https://soybase.org/snps/). The information on the 562 elite lines (153 southern elites, 313 northern elites, and 96 elites not classified as southern elites or northern elites) used for this study were listed in Table S1.

**Core Ideas**

- Two assays containing SoySNP3K and SoySNP1K single‐nucleotide polymorphisms (SNPs) and markers associated with soybean traits were developed.
- SNPs in the assays are highly polymorphic among US Elites and well cover genome haplotype blocks.
- The assays are valuable resource for soybean genetic research and breeding applications.
- These assays are commercialized and used by soybean researchers.

### 2.2 Selection of SNPs included in the 3K and 1K assays

The 3K SNPs were a subset of 6K SNPs, and 1K SNPs were a subset of 3K SNPs. The SNPs in the 6K were originally selected from 50K, which all have a high minor allele frequency (MAF) among the 14,183 *G. max* accessions and have generated high‐quality genotypic data for the germplasm and thousands of RILs (Song et al., 2020). The 3K SNPs were selected from each of the large *G. max* haplotype blocks with a size of ≥50 kb and ≥100 kb in the euchromatic regions and heterochromatic regions, the remaining SNPs were selected from the segments between haplotype blocks in heterochromatic and heterochromatic regions, respectively. These haplotype blocks were previously delimited based on the analysis of the 50K dataset containing 14,183 *G. max* genotyped with 42,509 SNPs (Song et al., 2020). The 1K containing 1252 SNPs were selected from the 3K panel with reduced number of SNPs in the same haplotype blocks based on the analysis of the 562 North American elites. The haplotype blocks in North American elites were identified by Song et al. (2015) using the software PLINK (Purcell et al., 2007). SNPs with MAF < 0.10 in the North American elites were also eliminated in the 1K assay. The density of selected SNPs in the euchromatin region versus the heterochromatin region is approximately 5:1 for both 3K and 1K, which is consistent with the 5:1 ratio of recombination rate/Mb in the two regions of the soybean genome (Song et al., 2013).

### 2.3 Estimation of polymorphic SNP number between two randomly selected accessions in northern and southern soybean elite lines

Numbers of pair‐wise polymorphic SNPs among 155 southern elites and 313 northern elites based on the 3K and 1K SNPs were calculated, respectively. The projected number of polymorphic SNPs between any pair of random accessions within southern and northern elites was calculated based on the average number of all pair‐wise polymorphic SNPs and the standard error of the pair‐wise polymorphic SNP numbers at 95% probability.

### 2.4 Determination of SNP genomic positions in soybean WGS assembly Wm82a4.1

Physical positions of 50K SNPs in Glyma1.01 and Wm82a2v1 WGS assemblies were determined previously (Song et al., 2016). The SNP positions in Wm82a4.1 were determined using the Blast software downloaded at NCBI site (https://blast.ncbi.nlm.nih.gov/doc/blast‐help/downloadblastdata.html#downloadblastdata). The 61‐bp sequence flanking each SNP (Song et al., 2013) was blasted to the Wm82a4.1 WGS downloaded at Phytozome (https://phytozome‐next.jgi.doe.gov/info/Gmax_Wm82_a4_v1). Then positions of the SNPs in Wm82a4.1 were extracted if their flanking sequence is 100% aligned to the WGS. The linkage map positions of SNPs in 50K were previously determined (Chen et al., 2022). Because the 1K and 3K SNPs are subsets of 50K SNPs, the genomic and linkage map positions of SNPs in both 3K and 1K were extracted from those of the 50K SNPs for the calculation of linkage map distance distribution of the SNPs in 3K and 1K.

## 3 RESULTS

### 3.1 SNPs in 3K and 1K

The 3K assay includes 1427 SNPs from each of the large *G. max* haplotype blocks with a size of ≥50 kb in the euchromatic regions and ≥100 kb in the heterochromatic regions, 59 SNPs and 1587 SNPs from the segments between haplotype blocks in heterochromatic and heterochromatic regions, respectively. The 1K consisting of 1252 SNPs was selected from 3K. Of these SNPs, a total of 1175 SNPs were from 1175 *G. max* haplotype blocks (Table S2). The 3K and 1K also contained 88 SNP markers that were either embedded in or associated with the genes controlling traits of interest, including growth habit, morphological features, flowering time, maturity, seed composition, abiotic stress tolerances, and disease resistance (https://www.agriplexgenomics.com/soybean‐community‐panel).

### 3.2 MAF and polymorphism rate of the SNPs in 3K and 1K assays

**FIGURE 1:**
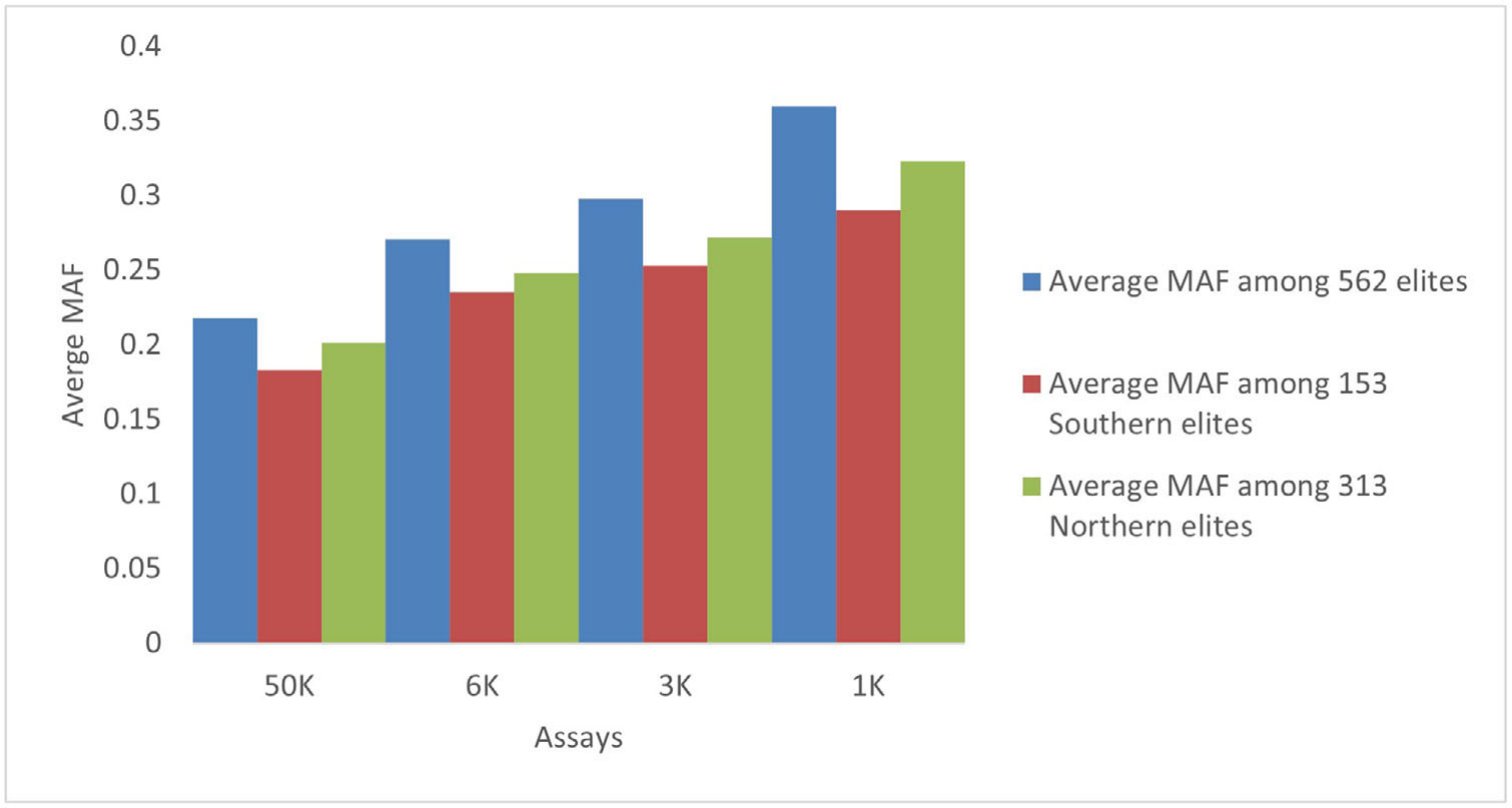
Average minor allele frequency (MAF) of single‐nucleotide polymorphisms (SNPs) in SoySNP50K (50K), SoySNP6K (6K), SoySNP3K (3K), and SoySNP1K (1K) based on 563 North American elite lines.

The average MAF of 3K SNPs among the 562 elite lines was 0.30, with the average MAFs of the southern and northern elites 0.25 and 0.27, respectively. In comparison, the average MAF of 1K SNPs among the 562 elites was 0.36, and the average MAFs of the southern and northern elites were 0.29 and 0.32, respectively. The average MAF of SNPs among elites was higher in 3K and 1K than 50K and 6K (Figure 1; Table S2), suggesting that selection of SNPs for inclusion in 1K and 3K significantly reduced SNPs with low MAF and retained SNPs with high MAF.

The percentage of SNPs with MAF < 0.05 among 562 elites was 5.9% in 3K and 0 in 1K, which were lower than that in 50K and 6K (Table 1). The percentage of SNPs with MAF < 0.05 in the southern elites was 9.97% and 5.22% in 3K and 1K, respectively. In comparison, the percentage of SNPs with MAF < 0.05 in the northern elites was 9.12% and 0.88% in 3K and 1K, respectively (Tables 2 and 3), which was also lower than the percentage in 50K and 6K. The relatively low percentages of SNPs with low MAF suggested that 3K and 1K assays could be used for both southern and northern germplasm. For 1K assay, the expected mean number of polymorphic SNPs between two random accessions was 456, ranging from 442 to 526 in the southern elites, and 509, ranging from 499 to 563 in the northern elites, at the 95% probability level. For 3K, the expected mean number of polymorphic SNPs in two randomly selected accessions was 1000, ranging from 967 to 1152 in southern elites, and 1063, ranging from 1042 to 1176 in the northern elites. The expected mean number of polymorphic SNPs among two random *G. max* accessions was 1331, ranging from 1021 to 1641 for 3K, and 544, ranging from 399 to 689 for 1K.

**TABLE 1:** Percentage of single‐nucleotide polymorphisms (SNPs) with a minor allele frequency (MAF) < 0.05 among 562 elite lines for each assay.

Assay ID	Number of monomorphic SNPs	Number of SNPs with MAF < 0.05	Total number of SNPs with MAF < 0.05	Total number of SNPs in the assay	Percentage of SNPs with MAF < 0.05
50K	3828	7439	11267	42,509	26.50
6K	4	609	613	6002	10.21
3K	0	181	181	3072	5.90
1K	0	0	0	1252	0

Abbreviations: 1K, SoySNP1K; 3K, SoySNP3K; 5K, SoySNP50K; 6K, SoySNP6K.

**TABLE 2:** Percentage of single‐nucleotide polymorphisms (SNPs) with minor allele frequency (MAF) < 0.05 among 153 southern elite lines.

Assay ID	Number of mono SNPs	Number of SNPs with MAF < 0.05	Total number of SNPs <0.05 MAF	Total number of SNPs in the assay	Percentage of SNPs with MAF > 0.05
50K	6251	7821	14,072	42,509	33.10
6K	105	793	898	6002	14.96
3K	21	285	306	3072	9.97
1K	0	65	65	1252	5.20

Abbreviations: 1K, SoySNP1K; 3K, SoySNP3K; 5K, SoySNP50K; 6K, SoySNP6K.

**TABLE 3:** Percentage of single‐nucleotide polymorphisms (SNPs) with minor allele frequency (MAF) < 0.05 among 313 northern elite lines.

Assay	Number of mono SNPs	Number of SNPs with MAF < 0.05	Total number of SNPs <0.05 MAF	Total number of SNPs in the assay	Percentage of SNPs with MAF < 0.05
50K	5439	7525	12,964	42,509	30.50
6K	80	592	672	6002	11.20
3K	14	266	280	3072	9.12
1K	0	11	11	1252	0.88

Abbreviations: 1K, SoySNP1K; 3K, SoySNP3K; 5K, SoySNP50K; 6K, SoySNP6K.

### 3.3 SNP allele sharing between northern and southern elites

Southern and northern elites shared the same major alleles for 709 SNPs in the 1K. Out of the 543 remaining SNPs where the major alleles differed between southern and northern elites, 406 SNPs had major alleles in the northern elites but had minor alleles in southern elites. In the 3K, southern and northern elites had the same major alleles for 2041 SNPs. Of the 1031 SNPs in which the major alleles differed between southern and northern elites, 793 SNPs had major alleles in the northern elites but were minor alleles in southern elites. Only 306 and 280 SNPs in 3K had MAFs < 5% in southern and northern elites, compared to 65 and 11 in 1K, respectively (Tables S1 and S2). The small number of SNPs from 3K and 1K with low MAF SNPs in northern and southern elites suggests that these assays should work well in both southern and northern breeding programs despite differences of allele frequencies between southern and northern elites.

### 3.4 Position of SNPs in Wm82a4.1 WGS assembly and distribution of SNPs on genetic linkage maps

**FIGURE 2:**
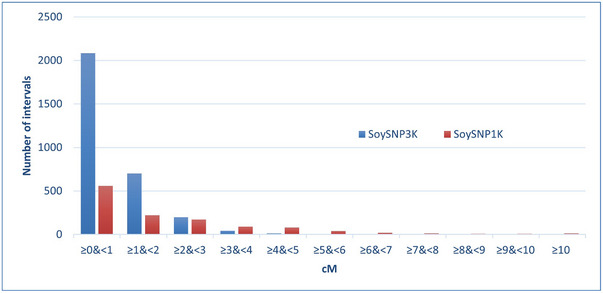
Distribution of single‐nucleotide polymorphism (SNP) marker intervals in SoySNP3K and SoySNP1K.

The positions of 50K SNPs, including 3075 SNPs in 3K and 1247 SNPs in 1K, were mapped to 20 chromosomes of the v4 assembly (Table S2), and the SNP number per chromosome ranged from 111 to 205 for 3K assay and 33 to 94 for 1K assay. Seven SNPs in 3K and five SNPs in 1K were not mapped to chromosomes but unanchored scaffolds (Table 4). Analysis of the inferred linkage map positions of these SNPs showed that average distance intervals between two adjacent SNPs in 3K and 1K were 0.82 and 1.94 cM, respectively, and 99.6% and 91.8% of the intervals between two adjacent SNPs in 3K in 1K were less than 5 cM, respectively. The number of large intervals in the genome is small (Figure 2). The results showed that SNPs included in the two assays were well distributed across the soybean genome.

**TABLE 4:** Single‐nucleotide polymorphism (SNP) number per chromosome based on the alignment of SoySNP1K (1K) and SoySNP3K (3K) SNPs to the Wm82a4.1 whole genome sequence assembly.

Chromosome	SoySNP3K	SoySNP1K
Gm01	127	62
Gm02	171	83
Gm03	133	53
Gm04	147	72
Gm05	146	53
Gm06	155	55
Gm07	160	61
Gm08	197	67
Gm09	146	66
Gm10	170	69
Gm11	131	33
Gm12	111	40
Gm13	185	68
Gm14	137	56
Gm15	174	90
Gm16	115	47
Gm17	142	61
Gm18	205	94
Gm19	182	72
Gm20	130	45
Scaffolds/unknown	8	5
Total	3072	1252

## 4 DISCUSSION

Soybean is a self‐pollinating crop, lack of recombination in the genome results in extensive linkage disequilibrium and large haplotype blocks, which are the basis for detecting associations between traits and markers in linkage analysis or genome‐wide association studies (Song et al., 2015). It is estimated that approximately 50% of the genomic sequence in the euchromatic and heterochromatic regions of the soybean genome is in haplotype blocks (Song et al., 2015). SNPs in the same haplotype block are most likely to give rise to the same segregation pattern, especially for RILs derived from biparental populations or germplasm accessions that share the same geographic origin. This is evidenced by the high redundancy of markers genotyped by sequencing methods in RIL populations of soybean and other crops (Poland et al., 2012; Punnuri et al., 2016; Qi et al., 2014), suggesting that trait and marker association detection, especially in RIL populations, does not require many markers, as long as the markers capture haplotype block variation. Soybean breeding involves parental selection, genomic prediction and selection, QTL mapping, and plant variety protection, assays with different number of markers are needed. The newly selected 3K and 1K assays along with 50K and 6K SNP assays constitute a series of nested marker panels that will meet these needs. The entire USDA germplasm collection and many populations from different laboratories have been genotyped using the 50K or 6K assays, new datasets generated from the 3K and 1K can be combined or compared with existing datasets to facilitate allele mining from germplasm collection and across breeding programs due to the nested nature of the markers in 50K, 6K, 3K, and 1K. In experiments where more markers are indeed required, in addition to genotype material with high density marker assays, parents of RIL populations can be genotyped with 50K or 6K, and then derived RIL population can be genotyped with 3K or 1K. Subsequently, the RIL can be imputed with 50K or 6K markers based on the 3K or 1K common markers in the population, and the current imputation method can impute with high accuracy (Chen et al., 2022).

In order to select the 3K and 1K SNPs to be included in the assays, factors required for successful assay design were considered, including high MAF in North American elites and *G. max*, capture of large haplotype block variations in the soybean genome, and reduction of SNPs with redundant allele profiles among population individuals. The assays will not only be useful for *G. max* accessions but also for southern and northern elite lines, even though southern and northern elites may have different population structures. There are some limitations for the assay applications and the number of markers in the 3K and 1K assays may be insufficient to fine map the gene/QTL underlying traits in a large RIL population derived from *G max* × *G. soja* cross via genetic linkage association analysis, because the size of linkage disequilibrium blocks is smaller in wild soybean than in cultivated soybean (Song et al., 2020).

The 3K assay has been commercialized by Illumina Inc., using beadchip assay technology, and 1K assay has been commercialized by AgriPlex Genomics, using targeted sequencing approach and a PlexSeq platform. Although 3K assay is on a beadchip platform, the SNP can also be used for developing targeted amplicon GBS assay in soybean (Mamanova et al., 2010; Niedzicka et al., 2016; Turner et al., 2009), if the array technology reaches its life span or costs more.

The 3K has been used to predict optimal cross combinations to accelerate genetic improvement of soybean seed yield (Miller et al., 2023b). The trend of the prediction accuracy across different statistical prediction models was consistent between 6K and 3K, although the prediction efficiency is slightly higher using the 6K than the 3K based on the analysis of the yield of 42 crosses. The 3K was also evaluated for its efficiency to predict yield and seed composition traits in 1500 inbred lines developed at University of Georgia. Similar pattern of prediction efficiencies were observed across different traits and prediction models between 6K or 3K assays, although in each case, the accuracy is slightly lower in 3K than 6K (Miller et al., 2023a).

The 1K SNP assay has been used to genotype over 10,000 soybean breeding lines provided by soybean breeders from Southern and Northern US. breeding programs, and > 87% of the SNPs were successfully genotyped, and > 94% of these SNPs were polymorphic within populations (Beni Kaufman, personal communication, 2024).

Since the 3K and 1K assays also include a number of trait markers reported or contributed by soybean communities, these assays will also aid in the evaluation and selection of breeding lines with desired traits. As more markers associated with the trait are identified in the future, they can be added to the 3K and 1K assays due to the flexibility of both beadchip assay and targeted sequencing approaches. In summary, 3K and 1K will be good lower cost tools that address soybean breeders' needs to characterize and select soybean breeding lines.

## AUTHOR CONTRIBUTIONS

**Qijian Song**: Conceptualization; formal analysis; funding acquisition; investigation; supervision; writing—original draft. **Charles Quigley**: Investigation; resources. **Ruifeng He**: Validation. **Dechun Wang**: Validation; writing—review and editing. **Henry Nguyen**: Validation; writing—review and editing. **Carrie Miranda**: Resources. **Zenglu Li**: Resources; writing—review and editing.

## CONFLICT OF INTEREST STATEMENT

The authors declare no conflicts of interests.

## Supporting information

Supplementary Table S1. Maturity groups and geographic origin of 562 North American soybean elites

Supplementary Table S2. 50K SNP genomic positions in Williams 82 V1, V2, and V4 whole genome sequence assemblies, inferred linkage map positions as well as MAF differences between Northern and Southern soybean elites.

## Data Availability

The SoySNP50K dataset used to calculate SNP MAF and delimit haplotype blocks is available at https://www.soybase.org/snps/. The list of elite accessions used in this study, SNP genetic linkage map positions, SNP physical locations in different WGS assemblies, and SNP MAFs of southern and northern elite accessions are listed in Supplementary Table S1 or S2.
